# Reduced Viability, Fertility and Fecundity in Mice Lacking the Cajal Body Marker Protein, Coilin

**DOI:** 10.1371/journal.pone.0006171

**Published:** 2009-07-09

**Authors:** Michael P. Walker, Liping Tian, A. Gregory Matera

**Affiliations:** 1 Department of Genetics, School of Medicine, Case Western Reserve University, Cleveland, Ohio, United States of America; 2 Departments of Biology and Genetics, Program in Molecular Biology & Biotechnology, Lineberger Comprehensive Cancer Center, University of North Carolina, Chapel Hill, North Carolina, United States of America; Brunel University, United Kingdom

## Abstract

**Background:**

Coilin is the signature protein of the Cajal body, a conserved nuclear organelle involved in multiple aspects of small ribonucleoprotein (RNP) biogenesis. Coilin is required for Cajal body homeostasis in both plants and animals. Mice lacking coilin are viable when the mutation is crossed to an outbred strain but only partially viable when crossed to inbred lines.

**Methodology/Principal Findings:**

In order to clarify this issue, we backcrossed the coilin deletion onto the C57BL6/J background for ten generations and then investigated the consequences of coilin removal on overall viability and reproductive success. We conclude that semi-lethal phenotype observed in mixed-background crosses is due to loss of the *Coilin* gene (or a very tightly-linked locus). Interestingly, coilin knockout embryos die relatively late in gestation, between E13.5 and birth. We show that the maternal contribution of coilin is not important for organismal viability. Importantly, coilin knockout mice display significant fertility and fecundity defects. Mutant males that escape the embryonic lethality display reduced testis size, however, both males and females contribute to the observed reduction in reproductive fitness.

**Conclusions/Significance:**

The evolutionary conservation of coilin from plants to animals suggests that the protein plays an important role, perhaps coordinating the activities of various RNA-processing machineries. Our observations are consistent with the idea that coilin functions to ensure robust organismal development, especially during periods of rapid growth.

## Introduction

Small nuclear ribonucleoproteins (snRNPs) are essential components of the spliceosome, required for proper pre-mRNA splicing [1]. SnRNP biogenesis is a complex pathway that begins in the nucleus with transcription of small nuclear RNA (snRNA) and subsequent export to the cytoplasm [2]. Once in the cytoplasm, the snRNAs form complexes with a core set of seven Sm proteins. The assembly of the heptameric Sm core is facilitated by the survival motor neuron (SMN) protein complex. This complex contains eight associated proteins, called Gemins, along with the spinal muscular atrophy disease gene product, SMN [3]. The addition of the Sm core serves as a signal for processing at both the 5′ and 3′ ends of the snRNA. Upon completion of these steps, the partially-assembled snRNP is imported back into the nucleus. Newly formed snRNPs enter the nucleus and first accumulate within nuclear structures called Cajal bodies[4], where additional RNP maturation steps are thought to take place [5], [6], [7], [8], [9], [10], [11], [12], [13], [14], [15].

The Cajal body was first characterized over one hundred years ago by the Spanish neurocytologist Santiago Ramón y Cajal, using silver staining. A large argyophilic nuclear body, often located in close proximity to the nucleolus was termed by Cajal the “accessory body.” These structures were later rediscovered numerous times and given different names [16], [17]. In 1999, these structures were re-named as Cajal bodies, in honor of their discoverer [18]. The Cajal body is a relatively large (0.2–1.0 µm in diameter) macro-molecular structure comprised of many different proteins and RNAs, most of which are also concentrated in other sub-nuclear domains. For example, Nopp140, fibrillarin and snoRNAs are found in Cajal bodies, but also accumulate in the nucleolus [19]. The SMN complex accumulates in Cajal bodies, but is also present in the cytoplasm and in twin structures, called Gemini bodies or gems [20], [21]. In contrast, coilin, which was first characterized through the use of autoimmune patient sera, is highly concentrated in the Cajal body and is diffusely localized throughout the nucleoplasm [22], [23]. Coilin has since become the primary molecular marker used to identify Cajal bodies in vertebrate cells.

Functional studies in vertebrates and invertebrates have shown that coilin is required for the formation of proper Cajal bodies [24], [25], [26], [27]. Notably, recruitment of the mammalian SMN complex to Cajal bodies is mediated by RG-rich residues within the coilin C-terminal domain [28], [29]. Murine *Coilin* is located on chromosome 11 and encodes a 61.8 kDa protein comprised of 568 amino acids; the protein is expressed in all tissues examined with a rather high concentration in brain and especially the testis [30]. Deletion of 85% of the coilin coding region, encompassing the C-terminal 486 aa has a profound effect on Cajal body formation in both adult tissues and embryonic fibroblasts derived from *Coil −/−* mice. Coilin knockout cells display at least three distinct types of “residual” structures [7], [25], reviewed in [20]. Normally, the components present in the residual bodies are located together with coilin in the Cajal body. Importantly, when exogenous coilin was expressed in *Coil −/−* cells, the residual structures disappeared and Cajal bodies were re-formed [25]. Thus the cellular phenotypes described in previous studies of mouse coilin knockout cells reveal that coilin is required for Cajal body formation and for recruitment of splicing snRNPs, modification guide RNAs, and the SMN complex [25], [28], [29], [31], [32]. Despite these advances, characterization of organismal phenotypes in coilin knockout mice has lagged behind.

Previously, we showed that loss of coilin was, somewhat surprisingly, homozygous viable when the mutation was maintained on an outbred CD-1 background [25]. Contrastingly, roughly half of the F1 homozygotes died when crossed onto inbred lines [25]. Because the heterozygous animals used for the intercrosses described above had only been backcrossed for a single generation onto their respective inbred strains, it was possible that the homozygous lethality we observed was due to a second site mutation in the ES cell line used in creation of the chimeric mice. In this study, we backcrossed the animals for ten generations onto the C57BL6/J background and then analyzed the progeny of heterozygous intercrosses. Furthermore, because the animals from the previous study [25] were genotyped at weaning (post-natal day 21, P21), the phenocritical phase was not established. Genotyping at embryonic day 13.5 (E13.5), P1 and P10 allowed us to restrict the time period of lethality to between E13.5 and P1. Finally, we show that *Coil −/−* mice display significant fertility and fecundity defects as compared to controls.

## Results

### Homozygous loss of coilin is semi-lethal

Previous observations showed that *Coil* −/− mice were significantly under-represented at weaning (P21) when first generation founder mice were crossed to either 129Sv/J or C57BL6/J inbred strains [25]. However, these mice were analyzed after only one generation of backcrossing. Thus the progeny contained 50% C57BL6/J and 50% 129Sv/J (from the ES cells used to create the mutation). Hence, the reduced number of *Coil −/−* mice observed at weaning could, in principle, be due to a second site mutation contained within the ES cell line used to create the knockout. In order to address this caveat, we backcrossed animals heterozygous at the *Coil* locus with wild-type C57BL6 mice (obtained from Jackson Laboratories) for ten generations, each time selecting for the *Coilin* deletion allele. Therefore, any remaining 129Sv/J alleles must be tightly linked to the *Coilin* gene. We then intercrossed heterozygotes and genotyped the progeny at four different developmental time points. As shown in Table 1, and consistent with previous results, we found that *Coil −/−* mice were significantly under-represented when genotyped at weaning (P21). In order to determine if the animals were dying early in development or later on, we genotyped mid-gestation embryos (E13.5) and found that the number of homozygous mutants did not significantly differ from the expected number (Table 1). Thus the animals must have died between E13.5 and P21. To narrow down this lethal window, we genotyped neonatal (P1) mice and found that their numbers were significantly reduced. Similar results were obtained for P10 animals (Table 1). Importantly, the ratios of *Coil* +/+ and +/− mice remained a relatively constant 1∶2. Thus, we conclude that roughly half of the coilin knockout mice died late in gestation (i.e. between E13.5 and birth), whereas the other half survived. The *Coil −/−* mice that survived to weaning showed no gross morphological or behavioral defects. The majority of the runts genotyped at weaning were *Coil* −/− mice, however, these animals were indistinguishable in size from their littermates after they reached sexual maturity (data not shown). These findings were somewhat surprising, given that analysis of mutations in other genes involved in snRNP biogenesis, such as *Smn*, *Gemin2* and *Zpr1*, each displayed early embryonic lethality [33], [34], [35].

**Table 1:** *Coil* −/− mice display reduced viability.

	E13.5			P1			P10			P21		
Genotype	+/+	+/−	−/−	+/+	+/−	−/−	+/+	+/−	−/−	+/+	+/−	−/−
Observed	19	38	18	27	66	15	21	58	11	55	114	26
Expected	18.8	37.4	18.8	27	54	27	22.5	45	22.5	48.8	97.4	48.8
p-value	0.99			0.018			0.008			0.001		

Heterozygous animals were intercrossed and the progeny were genotyped at E13.5, P1, P10 and P21. P-values were calculated by chi-square analysis for each developmental time point.

### Maternal contribution of coilin is not important for viability

The decreased viability of *Coil* −/− mice could be explained as a purely developmental defect, wherein the knockout mice are less fit than their littermates and, consequently, are more susceptible to late gestation arrest. Alternatively, the reduced viability of *Coil* −/− embryos might be due to a suboptimal uterine environment in the mother. To address this question, we compared the number of *Coil* −/− neonates born to heterozygous versus homozygous mutant females. *Coil* −/− females were mated with *Coil* −/+ males or *Coil* −/+ females were mated with *Coil* −/− males, ensuring that only homozygous mutant and heterozygous littermates were produced. Consistent with the reduced viability of the knockout animals, there were fewer homozygous mutant pups than heterozygotes (Table 2), but the difference in number was not as pronounced as that observed for the heterozygous intercrosses. Importantly, a Student's T-test comparing the results of the two groups in Table 2 found no significant differences. We therefore conclude that the absence of a maternal contribution of coilin in the oocyte has no significant effect on the viability of the progeny.

**Table 2:** Contribution of the uterine environment to neonatal viability.

	♀*Coil* −/−*X*♂*Coil* +/−		♀*Coil* +/−*X*♂*Coil* −/−	
Genotype	+/−	−/−	+/−	−/−
Observed	64	48	63	40
Expected	56	56	51.5	51.5
p-value	0.13		0.02	

Homozygous mutant animals (male or female) were crossed to heterozygous mates and the progeny were genotyped at P1. P values were calculated by chi-square analysis.

### Coil −/− mice display significant fecundity defects

**Figure 1:**
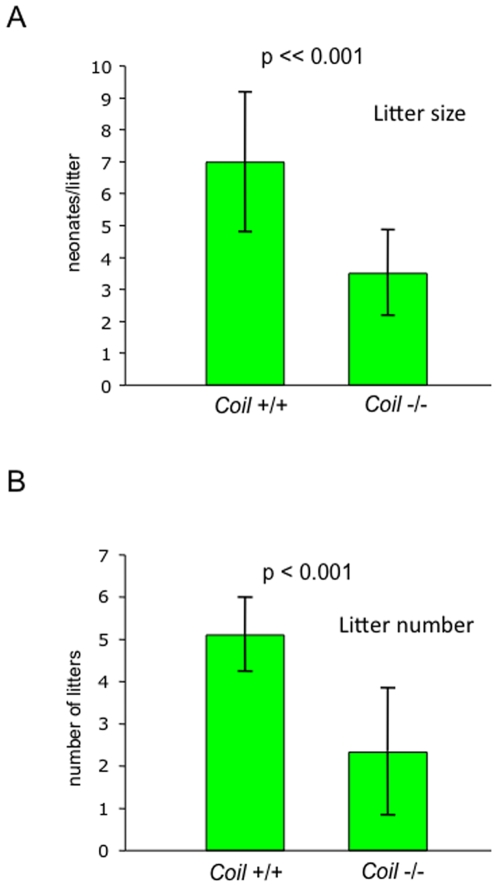
*Coil* −/− mice are reproductively less fit. The mean litter size (A), and the mean number of litters (B) were analyzed during a 6-month course of breeding. *Coil* −/− mice displayed reduced litter size and litter number compared to *Coil* +/+ controls, *p* = 2×10^−8^ and *p* = 3×10^−7^, respectively. P values were calculated by Student's T-test analysis.

Anecdotal observations indicated that *Coil* −/− mouse mating pairs were fertile, but that the litter sizes from these matings were smaller. To test whether *Coil* −/− mice display fecundity defects, we compared litter size and litter number over a six-month time period. Mating pairs were established from intercrosses of heterozygous mice. Wild-type and homozygous animals derived from these crosses were age-matched and the number of pups per litter was measured during the first six months after the females had reached sexual maturity (P42). We found that the mean litter size, 3.3±1.4, of *Coil* −/− mating pairs was significantly smaller than that of wild-type mating pairs, 6.9±2.2 (Figure 1A). Importantly, the average litter size of the wild-type C57BL6/J mating pairs was in good agreement with published data for C57BL6/J mice [36]. Thus, coilin knockout females have fewer pups per litter than do their wild-type counterparts.

In addition to the nearly two-fold reduction in litter size, homozygous mutant mating pairs also appeared to produce fewer litters overall. An analysis of litter number revealed that wild-type mating pairs produced significantly more litters over a six month period than did the *Coil* −/− mating pairs. On average, *Coil* +/+ females gave birth to 5.3±1.1 litters over the first 6 months of breeding, whereas *Coil* −/− females gave birth to only 3.0±0.7 litters over the same time period (Figure 1B). Thus the reproductive output of *Coil* −/− mice is significantly less than that of wild-type C57BL6/J mice.

### Male and female Coil −/− mice contribute equally to their reduced fecundity

**Figure 2:**
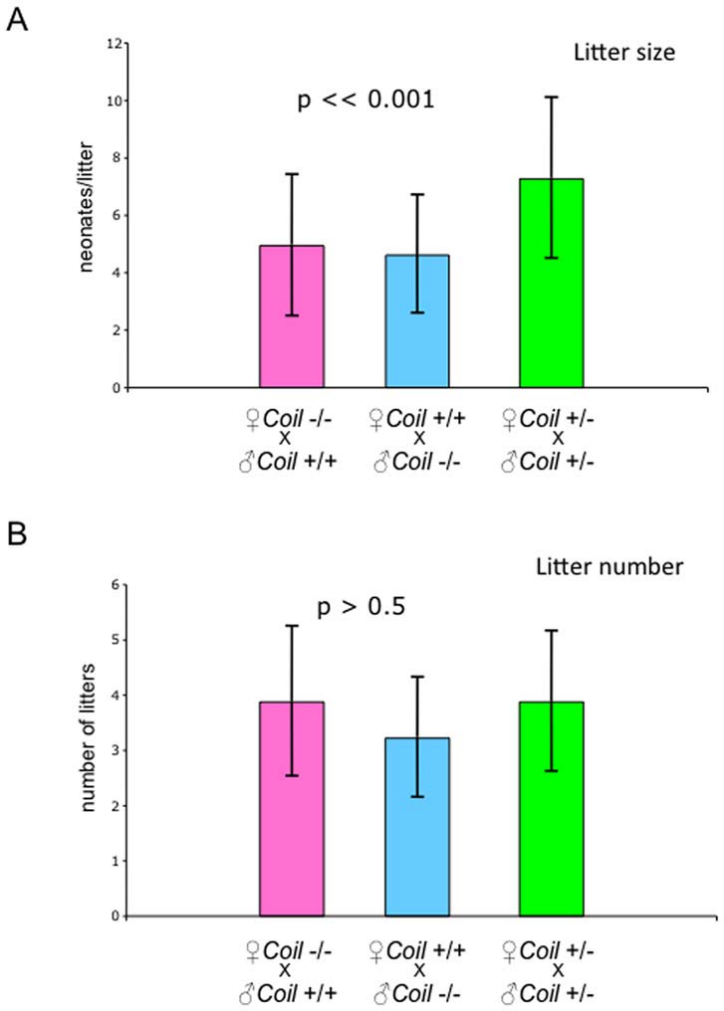
Reciprocal mating crosses show that male and female *Coil* −/− mice contribute to reduced reproductive output. The mean litter size (A), and the mean number of litters at 4 months were analyzed, (B). (*Coil* −/− female)/(*Coil* +/+ male) and (*Coil* +/+female)/(*Coil* −/− male) mating pairs were compared to control heterozygous mating pairs. In (A), mating pairs that contain a male or female *Coil* −/− mouse displayed smaller litter sizes compared to the control mating pair; *p* = 4×10^−5^. Litter numbers in panel (B) were not significantly different. P values were calculated by ANOVA testing.

Reciprocal mating pairs (i.e. *Coil* −/− females and wild-type males as well as wild-type females with *Coil* −/− males) were established to determine whether gender contributed to the reduced fecundity observed in Figure 1A,B. Again, we measured litter size and litter number over time. As a control, heterozygous mating pairs were also analyzed. The mean litter size was not significantly different between the reciprocal mating pairs (Figure 2A). However, each produced significantly smaller litters (4.9±0.8 and 4.6±0.9, respectively) than did the control heterozygous mating pairs (7.2±0.8; Figure 2A). Males and females therefore have the same influence on reduced litter size observed in homozygous intercrosses (Figure 1A).

Finally, we measured the number of litters derived from these reciprocal matings, as compared to the heterozygous intercrosses over a period of four months and found no significant difference in litter number among the three mating schemes (3.88±0.5, 3.22±0.7 and 3.88±1.3 Figure 2B). These results show that while litter size is affected, the reciprocal mating pairs are able to produce normal amounts of litters during this shorter time period.

Because the progeny from the reciprocal matings described in Figure 2A were genotypically identical (i.e. they were heterozygous), the smaller litter sizes we observed must be due to significantly reduced fecundity and not from reduced viability of the neonates. Moreover, these experiments represent a better test of the reproductive fitness of the *Coil* −/− females than are the results described in Table 2, because all of the progeny in these crosses are heterozygotes. Thus we conclude that the uterine environment of the *Coil* −/− female does not contribute significantly to the fitness of the progeny. Rather, the genotype of the progeny is the determining factor. Taken together, these data show that male and female *Coil* −/− mice contribute equally to the reduced fecundity phenotype.

### Reduction in testis size in Coil −/− mice

**Figure 3:**
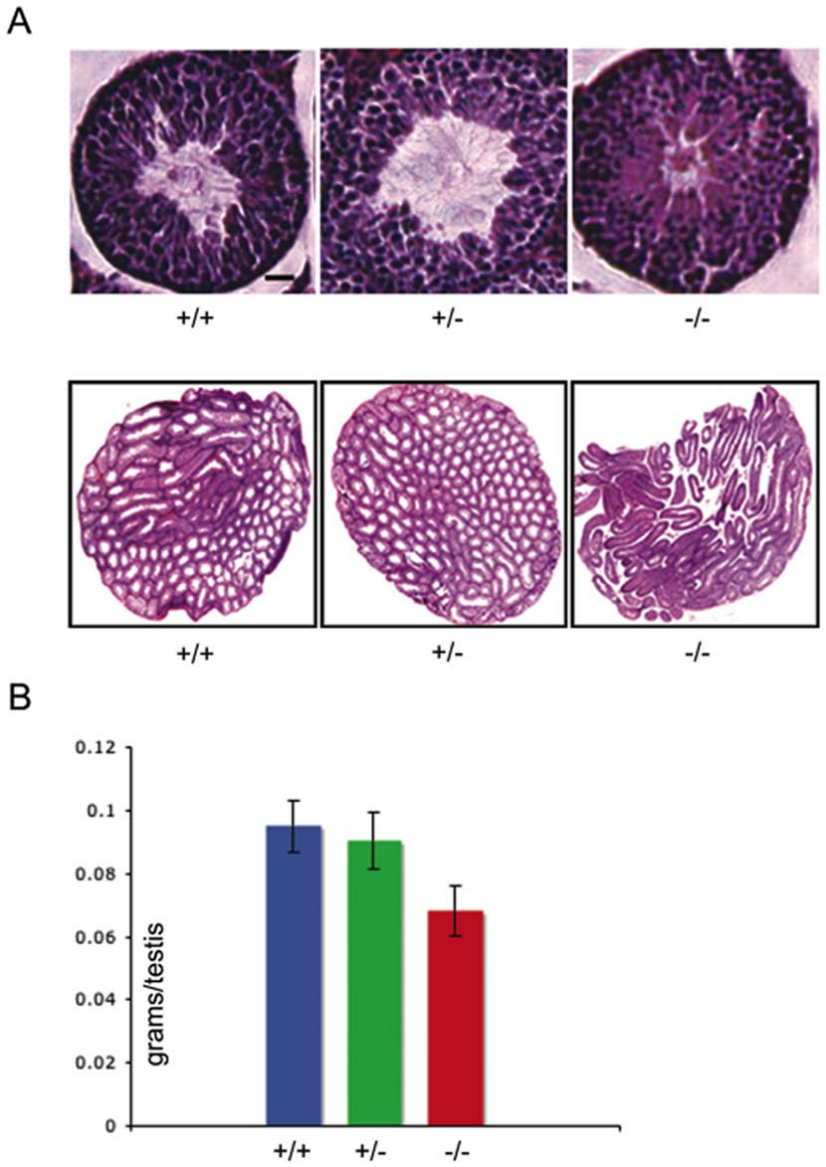
Coil −/− mice have reduced testis size. Testis sections were made and H & E stained from young adult male mice (56 days old; A). In the top panels individual seminiferous tubules were analyzed for any gross abnormalities; scale bar = 100 µm. Excised testes *of Coil −/−, +/−* and +/+ mice were individually weighed (B). Male *Coil −/−* mean testis weight is significantly lower than control littermates; *p*≪0.0001.

**Figure 4:**
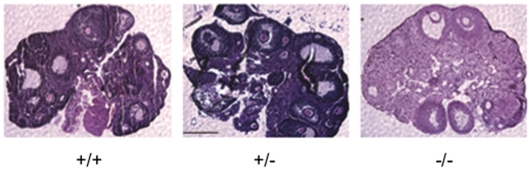
Analysis of *Coil −/−* ovaries. Ovaries were removed from P42, sexually mature, females and cross sections were H & E stained. No obvious morphological abnormalities were detected among the genotypes. The parallel lines observed are artifacts from preparation of the tissue samples. The scale bar in the center panel is 2 mm.

Finally, we wanted to determine if obvious germline defects were the cause of the reduced fecundity observed above. Male mice were sacrificed at sexual maturity, P56, and their testes were excised for examination. We first examined cross sections of *Coil +/+*, *+/−* and *−/−* mice and observed no gross morphological differences (Figure 3A). However, we did observe a highly significant difference in mean testis weight. *Coil +/−* and *+/+* mice had fairly normal sized testes at 0.091±0.01 g and 0.095±0.01 g, respectively. By contrast, *Coil −/−* litter mates had smaller testes, weighing significantly less on average, at 0.068±0.01 g (Figure 3B). We also examined ovaries from P42 mutant females and compared them to their control littermates. No obvious differences were detected in the *Coil −/−* ovaries. In fact, ovaries from *Coil −/−* mice displayed mature oocyte follicles, similar to those of their *Coil +/+* and *+/−* siblings (Figure 4). Thus, loss of coilin does not significantly alter reproductive tissues, but does result in reduced testis size, which could contribute to their reduced reproductive fitness.

## Discussion

We have shown that *Coil* −/− mice display significant viability defects. The lethality manifests late in gestation, sometime between E13.5 and birth. However, the prenatal lethality is incompletely penetrant, as a fraction of the homozygous animals are viable, demonstrating that coilin is not an essential protein in mice. We also show that reduction or absence of coilin in the pregnant female does not contribute to the semi-lethal phenotype. Our observations are consistent with the idea that coilin function is necessary to ensure robust organismal development, especially during periods of rapid growth (e.g. late in gestation). Interestingly, *in silico* modeling experiments (based on *in vivo* measurements) have shown that concentration of snRNPs into Cajal bodies greatly facilitates assembly of U4/U6 di-snRNPs [37]. Obviously, the beneficial effects of Cajal bodies on “diffusion and capture” reactions need not be limited to the annealing of the U4/U6 di-snRNPs. Thus we view coilin as a kind of “efficiency” factor [38]. Indeed, the evolutionary conservation of coilin from plants to animals suggests it plays an important role, perhaps coordinating the activities of various RNA-processing machineries, allowing them to come together in one subnuclear locale [20], [39], [40].

In addition to the semi-lethality, coilin knockout mice also display reduced litter size and litter number, compared to wild-type controls. Sub-optimal germline development could contribute to the observed reduction in litter number. For example, *Coil* −/− males have smaller testes, which could reduce or delay sperm production. Conversely, the mutant females might produce fewer (mature) oocytes that are capable of being fertilized. Whatever the reason for the reduced reproductive output, it is clear that both males and females contribute to the smaller litter size, as the number of neonates per litter was low, irrespective of the gender of the homozygous mutant parent (Fig. 2A). The contribution to this effect may well be additive. These experiments demonstrate that coilin is not essential for fertility, but is needed for optimal reproductive fitness and development. Clearly, the loss of coilin would not be tolerated in a wild population of mice. Such animals would be less reproductively fit compared to their wild-type counterparts and would be rapidly out-competed.

On a molecular level, it is tempting to speculate that loss of coilin's interaction with SMN (and the concomitant failure to recruit SMN to Cajal bodies) might contribute to the reduced reproductive fitness of the coilin knockout. When coilin is deleted, the “residual” Cajal bodies lose their contact with the SMN complex [25], [28]. Because SMN is thought to be essential for snRNP assembly and recycling in vivo [3], [21], the lack of interaction between SMN and coilin in the nucleus might result in a diminished capacity for RNP assembly, which could have downstream effects on development and gametogenesis. Additional experiments will be required in order to test this hypothesis.

## Materials and Methods

### Animal care and genotyping

All animal protocols used in these studies were approved by the Case Western Reserve University Institutional Animal Care and Use Committee (IACUC). Mice were housed in microisolator cages in a barrier facility with an air shower entrance or in a specific pathogen-free facility. Mating cages typically consisted of one *Coil +/−* male and two *Coil+/−* females. Once pregnancy was detected, animals were put into separate cages and progeny were collected at P1, P10 or P21; these females were subsequently placed back into the mating cage for further mating. Approximately 2–3 mm of tail clipping from individual neonates was placed into a 1.5 ml microfuge tube and DNA extraction was carried out using the High Pure PCR Template Preparation Kit (Roche) following the manufacturers protocol. Genotyping by multiplex PCR analysis (Fischer Scientific) was carried out with the following three primer scheme: Forward primer, 5′-AAAGCAAGGTCAGACTATCGTTCC-3′; neo-reverse, 5′-TTTGCCAAGTTCTAATTCCATCAG-3′; coilin reverse, 5′-TTCACGTGGCTGCTTTGTTTTATC-3′.

### Embryo extraction

Individual heterozygous females were placed in a cage with isolated heterozygous males overnight. Early the next day these females were checked for plugs to ensure that mating had taken place. Plugged females were then housed in a cage until embryonic day 13.5 when they were then sacrificed by cervical dislocation. Embryos were excised from the uterus and individuals were carefully removed from the embryonic sac. Embryos were then washed 3× in ice cold 1XPBS (10×PBS: 580 mM Na_2_HPO_4_, 170 mM NaH_2_PO_4_•H_2_O, 680 mM NaCl) to ensure all maternal tissues were removed. Genotyping of PCR products from tissues derived from the limb of the embryo was carried out as described above.

### Histology of testes & ovaries

All mice were humanely euthanized according to protocols set forth by the Institutional Animal Care and Use Committee (IACUC) and CWRU Animal Resource Center (ARC). Testes or ovaries were excised from sacrificed mice, fixed in 10% formalin and 10 µm transverse sections were stained with hematoxylin and eosin.

### Statistical analysis

Chi-sqaure, student's T-test and ANOVA were performed using Smith's Statistical Package (SSP), version 2.75 for MAC OS X.
